# Hepatic Haemangioma Mimicking an Abscess in a Young Female: An Atypical Presentation of SLE

**DOI:** 10.7759/cureus.97518

**Published:** 2025-11-22

**Authors:** Azeem Faisal, Beth Turnpenny

**Affiliations:** 1 Geriatrics, Fairfield General Hospital, Bury, GBR; 2 Internal Medicine, Fairfield General Hospital, Bury, GBR

**Keywords:** hepatic abscess, liver haemangioma, pyrexia of unknown origin (puo), systemic autoimmune disease, systemic lupus erythematosus

## Abstract

This case describes a 33-year-old woman who presented with progressive weight loss, fever and fatigue, and was initially treated as a pyrexia of unknown origin. Computed tomography (CT) findings contributed to diagnostic momentum and suggested a hepatic abscess. Initial blood tests showed anaemia and leukopenia. Human immunodeficiency virus (HIV), hepatitis, *Treponema* and malaria screens were negative. Various antibiotics were trialled with no significant improvement in the clinical picture. A magnetic resonance imaging (MRI) of the liver excluded a hepatic abscess and instead confirmed a hepatic haemangioma. An autoimmune screen showed serology that was overall associated with systemic lupus erythematosus (SLE). The patient was promptly commenced on steroids and clinically improved. In this case report, we review the current literature around SLE and its association with hepatic haemangiomas. Key messages from this case include considering early autoimmune screens for atypical presentations and correlating radiological features with clinical findings.

## Introduction

This case report explores an atypical presentation of systemic lupus erythematosus (SLE), highlighting the importance of a broad range of differential diagnoses and preventing diagnostic momentum, a form of cognitive bias where initial diagnoses are accepted without challenge or review. Throughout this case, a prolonged focus was maintained on infectious causes, with extensive microbiology investigations and consultations. Computed tomography (CT) findings directly led to diagnostic momentum and ultimately delayed the correct diagnosis and prompt treatment. It is essential to remember that if symptoms do not correlate with the imaging findings or do not improve with standard treatments, then the clinical picture should be revisited, and alternative diagnoses should be considered.

## Case presentation

A 33-year-old Black woman (originally from South Africa) presented to the emergency department as an amber-standby, a term used in our local hospital to indicate that urgent assessment is required but not immediate resuscitation. An ambulance was called by the patient due to severe body aches and fever. She was triaged as a suspected sepsis. She described being generally unwell for eight months with unexplained weight loss of ~12.7 kg, fever and worsening appetite. No other infective/localising symptoms were reported. She reported returning from Spain three months prior, where the whole family had a diarrhoeal illness.

Past medical history of significance included psoriatic arthritis, for which the patient had previously been on methotrexate but stopped in 2021 as she was attempting to conceive. The patient had also recently had a lobar pneumonia nine months prior, but recovered well. An incidental liver haemangioma was found at that time on a CT of the thorax, abdomen and pelvis (CT TAP). A penicillin allergy, resulting in lip swelling, was also noted.

Observations on arrival included blood pressure of 104/69 mmHg, temperature of 38°C, heart rate of 107 beats per minute, respiratory rate of 20 breaths per minute and 100% SpO2 on room air. Examination was unremarkable aside from the patient appearing cachectic and underweight. Initial blood tests are recorded in Table 1.

**Table 1:** Blood tests on admission to the hospital ALT: alanine aminotransferase, ALP: alkaline phosphatase, CRP: C-reactive protein, ESR: erythrocyte sedimentation rate, eGFR: estimated glomerular filtration rate, HCG: human chorionic gonadotropin, HIV: human immunodeficiency virus

Laboratory tests	Result	Reference range
Haemoglobin	69 g/L	115-165 g/L
White blood cell count	2.6 × 10^9^/L	4-11 × 10^9^/L
Sodium	130 mmol/L	133-146 mmol/L
Potassium	4.5 mmol/L	3.5-5.3 mmol/L
Bilirubin	5 umol/L	0-20 umol/L
ALT	24 u/L	7-40 u/L
ALP	88 u/L	30-130 u/L
Albumin	36 g/L	35-50 g/L
CRP	36 mg/L	0-9.9 mg/L
ESR	129 mm/hour	5-15 mm/hour
eGFR	>90 mL/min/1.73 m^2^	> 60 mL/min/1.73 m^2^
HCG	<2.0 IU/L	1.5-4.2 IU/L
Hepatitis screen	Negative	N/A
HIV screen	Negative	N/A
Treponema antibodies	Negative	N/A
Malaria screen	Negative	N/A
Aerobic and anaerobic blood cultures	Negative	N/A

The patient was initially treated as a pyrexia of unknown origin, commenced on intravenous (IV) broad-spectrum antibiotics and transfused two units of blood. Lymphoma was also a differential diagnosis.

A CT TAP was performed two days into admission, with the indication of suspected lymphoma due to anaemia, fever, night sweats, pyrexia of unknown origin and a low white cell count.

**Figure 1:**
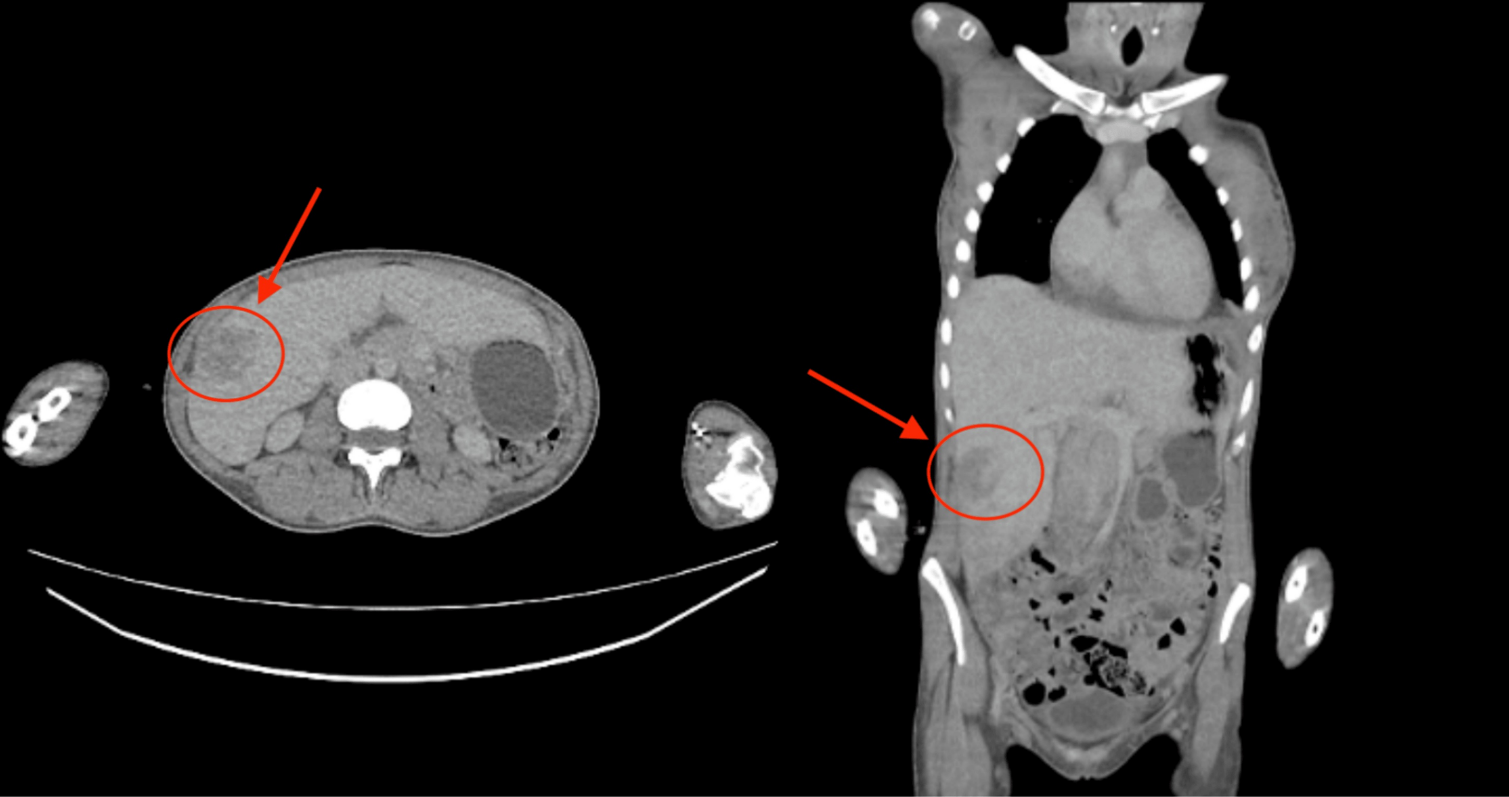
CT images showing a hepatic lesion within the right liver lobe CT: computed tomography

The CT suggested a 3.1 cm hepatic lesion within segment 5 (Figure 1), with advice that, given the patient’s symptoms, it should be suspicious for a hepatic abscess, but a malignant lesion could also not be excluded. Non-specific inflammatory changes in the lung were noted, along with a small pericardial and pleural effusion with axillary lymphadenopathy.

Following the CT TAP, both microbiology and infectious diseases were consulted, advising amoebic serology and magnetic resonance imaging (MRI) of the liver. Various antibiotics were commenced during this period, with no significant response. Antibiotic recommendations from microbiology included glycopeptides (vancomycin), nitroimidazoles (metronidazole) and aminoglycosides (gentamicin) to cover gram-positive, gram-negative and anaerobic bacteria. The patient remained intermittently febrile and tachycardic throughout the admission.

**Figure 2:**
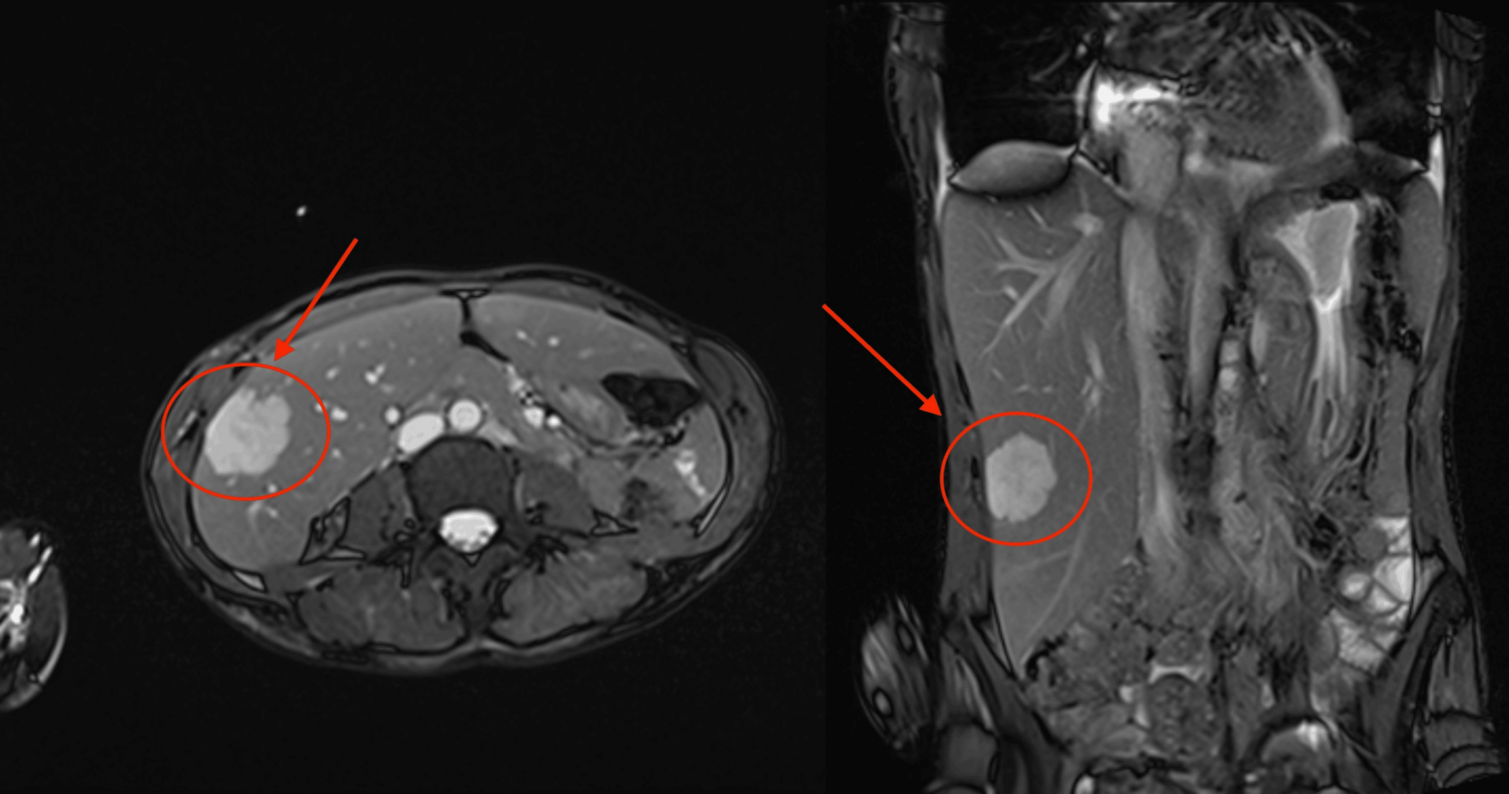
Liver MRI images further characterising the hepatic lesion as a benign haemangioma MRI: magnetic resonance imaging

An ultrasound of the abdomen was performed five days later, which characterised the lesion as 44 mm × 36 mm × 39 mm, with no obvious vascularity on Doppler. Given the absence of liver disease or previous malignancy, it was suggested to represent a benign haemangioma. An MRI of the liver (Figure 2) was subsequently performed, revealing a cavernous haemangioma in segment 5 and a further smaller subcapsular haemangioma in segment 7, and specifically, no abscess was seen. Based on this information, a biopsy was not advised. Antibiotics were also stopped at this point due to no convincing evidence of infection.

The results of the autoimmune screen were available following imaging (Table 2) and revealed positive anti-nuclear antibodies (ANAs), raised double-stranded DNA (dsDNA) antibodies and low C3 and C4 levels, overall serology that was associated with SLE.

**Table 2:** Autoimmune screen conducted in the hospital

Laboratory tests	Result	Reference range
Anti-nuclear antibody	Positive	N/A
Double-stranded DNA antibodies	126 IU/mL	0.0-9.9 IU/mL
C3 complement	0.57 g/L	0.75-1.65 g/L
C4 complement	0.11 g/L	0.14-0.54 g/L

Rheumatology input was sought following the autoimmune screen and imaging. It was advised to treat this as a new diagnosis of SLE and commence 40 mg of prednisolone daily until an urgent outpatient rheumatology review. They also advised that a lymph node biopsy was not indicated.

The patient reported symptomatic improvement following commencement of steroids, with both tachycardia and fever resolving. She was discharged once fever-free for 24 hours. Since discharge, the patient has had several rheumatology consultations. Methotrexate was recommenced for long-term immunosuppression, and the patient has reported overall improvement following commencement.

## Discussion

Systemic lupus erythematosus (SLE)

Systemic lupus erythematosus (SLE) is an autoimmune condition with a spectrum of clinical manifestations, ranging from mild mucocutaneous involvement to severe life-threatening multi-organ disease [1]. The prevalence of SLE disproportionately affects young Black women, with rates nearly five times higher than White patients and a female-to-male ratio of 9:1 [2]. Clinical features of SLE can be vague. More than 90% of patients with SLE will often present with constitutional symptoms (fatigue, fever, malaise and weight loss) [1]. More than 80% of patients will present with cutaneous and musculoskeletal involvement (ranging from mild to severe arthralgia and disability) [1].

The exact pathophysiology of SLE is complex and unclear. It is believed that a variety of immunological mechanisms are the cause (immune complex deposition and autoantibody formation resulting in inflammatory tissue injury), all influenced by environmental, hormonal and genetic factors [1,2].

Diagnosing SLE can be challenging given the vague presentation of symptoms and non-specific examination findings. Several biomarkers can be used to assist in the diagnosis. Of these, anti-nuclear antibodies (ANAs) have a high sensitivity and can be used first-line, but have a low specificity. In comparison, both anti-dsDNA and anti-Smith antibodies are highly specific for SLE but have a lower sensitivity: titres of antibody correlate closely to disease activity and so are more prone to fluctuate [2]. Low levels of C3 and C4 can be seen during flares, indicating complement consumption given the immune-mediated pathophysiology [1].

Low C3 or C4 levels, alongside a positive ANA, can be highly specific for SLE (94.3%), but this specificity significantly increases (97.6%) when a positive ANA is combined with both a low C3 and C4 [2].

Management of SLE is complex, requiring a multi-disciplinary approach to control the disease and maintain remission without relapses. Typically, steroids are used to induce remission in the acute stages, with disease-modifying anti-rheumatic drugs (DMARDs) such as hydroxychloroquine used to maintain remission [2]. Given the wide spectrum of disease, some individuals may also require cytotoxic agents and immunosuppressants to achieve remission [1]. General lifestyle advice, such as following a healthy diet and frequently exercising, can help to manage symptoms and reduce complications. Safe sun exposure with appropriate photoprotection (garments and sun protection factor 50 sun cream) and subsequent vitamin D supplementation is also vital to prevent flares [3].

Hepatic haemangiomas

Hepatic haemangiomas are common benign liver tumours, resulting from vascular malformations through abnormal angiogenesis [4]. Usually occurring in women between the ages of 30 and 50, hepatic haemangiomas are often single lesions and are typically found within the right hepatic lobe [5]. Growth of haemangiomas is rapid in young patients, with haemangiomas > 10 cm classed as giant haemangiomas. Growth rate tends to decrease with age and as haemangiomas increase in size [6,7].

Most haemangiomas are asymptomatic, but as size increases, they can cause non-specific symptoms of abdominal pain/discomfort [4]. Whilst the majority are asymptomatic, giant hepatic haemangiomas can lead to a rare complication known as Kasabach-Merritt syndrome (KMS). KMS, albeit rare, is a potentially life-threatening condition where a vascular tumour traps and destroys platelets and clotting factors, creating a cycle of bleeding and clotting that drives a consumptive coagulopathy, which can lead to fatal bleeding [7].

Ultrasound remains the first-line investigation of choice due to ease of accessibility. CT is slightly more sensitive (98.3%) but overall has a lower specificity (55%) when compared to all the other methods of imaging [4]. MRI remains the optimum method of imaging with both a high sensitivity (90%-100%) and specificity (91%-99%) of detecting hepatic haemangiomas [4]. Needle aspiration and biopsy are not recommended due to a high risk of haemorrhage and mortality, with a relatively low diagnostic yield [5].

The vast majority of haemangiomas will either be small or asymptomatic, and so do not require treatment [4]. Progression for hepatic haemangiomas varies; some will undergo degenerative and regressive changes (thrombosis, necrosis and fibrosis), but most will increase in size over a patient’s life [4,6].

Treatment is typically reserved for large, symptomatic haemangiomas or the presence of KMS [6]. Aside from surgical removal, other non-surgical options include transcatheter arterial embolisation (controlling acute bleeds or reducing the size of haemangiomas prior to surgery) or radiofrequency ablation (inducing thermal damage to promote thrombosis) [4].

SLE and its association with hepatic haemangiomas

SLE is known to affect multiple organs and systems. Hepatic involvement is typically subclinical with asymptomatic elevation of liver enzymes. However, a wide spectrum of liver disease has been reported within the literature, including portal changes, lobular changes, fibrosis and vascular changes [8].

SLE is associated with a higher prevalence of hepatic haemangiomas. One study found that patients with SLE were five times more likely to have hepatic haemangiomas when compared to their healthy control subjects (prevalence of 54.2% versus 14%, respectively) [9]. Currently, there is no clear understanding as to why SLE may predispose patients to hepatic haemangiomas. It has been theorised that haemangioma formation is due to unregulated angiogenesis, which can be promoted by oestrogen and other angiogenic factors, both of which can have increased levels in SLE [9].

## Conclusions

Autoimmune conditions can present vaguely. For younger patients, and particularly women of childbearing age, there should be a high degree of suspicion and a broad list of differentials for unexplained symptoms, with consideration for an early autoimmune screen. Not all pyrexias of unknown origin are infection-driven; therefore, it is important to avoid diagnostic momentum and correlate radiological features with clinical findings.

Our case highlights the challenges behind clinical reasoning and the importance of avoiding diagnostic momentum when evaluating persistent fever and systemic symptoms. Although this patient had a known hepatic haemangioma, it was re-investigated as a possible abscess based on initial imaging findings and her triage as a suspected sepsis. The lack of any significant improvement in empirical antibiotics should have prompted early consideration of alternative diagnoses. It is likely that our patient’s clinical picture reflected inflammation around the haemangioma, ultimately driven by uncontrolled SLE. Once infection was excluded and the appropriate specialties consulted, prompt corticosteroid therapy resulted in rapid clinical improvement.
